# N‐glycan in cockroach allergen regulates human basophil function

**DOI:** 10.1002/iid3.145

**Published:** 2017-02-20

**Authors:** Danh C. Do, Shuang Yang, Xu Yao, Robert G. Hamilton, John T. Schroeder, Peisong Gao

**Affiliations:** ^1^ Division of Allergy and Clinical Immunology Johns Hopkins University School of Medicine Baltimore Maryland USA; ^2^ Department of Pathology Clinical Chemistry Johns Hopkins University School of Medicine Baltimore Maryland USA; ^3^ Institute of Dermatology Chinese Academy of Medical Sciences and Peking Union Medical College Nanjing China

**Keywords:** C‐type lectin receptor, Cockroach allergen, Glycan

## Abstract

**Introduction:**

Cockroach allergen exposure elicits cockroach sensitization and poses an increased risk for asthma. However, the major components in cockroach allergen and the mechanisms underlying the induction of cockroach allergen‐induced allergy and asthma remain largely elusive. We sought to examine the role of cockroach‐associated glycan in regulating human basophil function.

**Methods:**

N‐linked glycans from naturally purified cockroach allergen Bla g 2 were characterized by MALDI‐TOF mass spectrometry. Binding of cockroach allergen to serum IgE from cockroach allergic subjects was determined by solid‐phase binding immunoassays. Role of cockroach associated glycan in histamine release and IL‐4 production from human basophils was examined. Expression of C‐type lectin receptors (CLRs) and their role in mediating glycan‐uptake in the basophils was also investigated.

**Results:**

MALDI‐TOF mass spectrometric analysis of N‐glycan from Bla g 2 showed complex hybrid‐types of glycans that terminated with mannose, galactose, and/or N‐acetyl glucosamine (GlcNAc). Deglycosylated Bla g 2 showed reduced binding to IgE and was less capable of inducing histamine release from human basophils. In contrast, N‐glycan derived from Bla g 2 significantly inhibited histamine release and IL‐4 production from basophils passively sensitized with serum from cockroach allergic subjects. An analysis of CLRs revealed the expression of DC‐SIGN and DCIR, but not MRC1 and dectin‐1, in human basophils. Neutralizing antibody to DCIR, but not DC‐SIGN, significantly inhibited Bla g 2 uptake by human basophils. A dose‐dependent bindings of cockroach allergen to DCIR was also observed.

**Conclusions:**

These observations indicate a previously unrecognized role for cockroach allergen‐associated glycans in allergen‐induced immune reactions, and DCIR may play a role in mediating the regulation of glycan on basophil function.

## Introduction

Cockroaches are a potent source of allergen that is known to induce sensitization and drive allergic respiratory symptoms 1, 2, 3, 4, 5, 6. Studies in children and adults from low‐income urban populations in the United States have demonstrated that cockroach allergen is present in 85% of inner‐city American homes. The prevalence of cockroach allergy in the United State ranges from 17% to 41% 1, 2, 7. However, the major immunogenic components and the mechanisms regarding allergic sensitization to cockroach allergen remain elusive.

Physiological extracts of cockroach have been widely used for years in the diagnosis of cockroach allergy by means of skin testing and cockroach allergen‐specific IgE serology. They have also been used to establish an experimental mouse model of allergic diseases 8, 9, 10. Importantly, immunotherapy for cockroach allergy has shown the promise as a treatment strategy with improved immune‐modulatory and clinical effects in a limited number of trials 11, 12, 13, 14. This highlights the importance of identifying all major allergenic components in allergens and understanding the mechanisms of cockroach‐induced allergy as well as developing therapeutic strategies. So far, a number of cockroach allergens have been identified, sequenced, purified, and produced as biologically active recombinant proteins. These include nine German (Bla g 1–8 and Bla g 11) and nine American cockroach allergens (Per a 1–3, Per a 6–7, and Per a 9–12) 6. These characterized cockroach allergens have led to an improvement in knowledge of the structure and function of cockroach allergens, and are crucial for the development of improved reagents for both diagnosis and therapy. However, the contribution of other potential virulence factors (i.e. macromolecules such as lipids and carbohydrates) that could be released by the cockroach may also contribute to the development of asthma.

Glycans are sugar notifications attached to glycoproteins and glycolipids. Recent studies have suggested that glycans may be crucial in allergen‐induced allergic responses 15, 18, 19. More specifically, glycans from *Fasciola hepatica* have been shown to modulate the host immune response and TLR‐induced maturation of dendritic cells 16. Complex carbohydrates have been considered potent inducers of Th2 responses, and carbohydrate antigens can stimulate the production of different isotypes of glycan‐specific antibodies 17. In particular, surface epitopes mapped from Bla g 2 demonstrated the presence of a surface carbohydrate moiety 18, 19 and the removal of this moiety by nucleotide point mutation significantly reduces IgE binding, IL‐13 production, and increased levels of IL‐10 20, 21. Furthermore, glycan on allergens may be directly involved in the uptake of allergens by carbohydrate lectin receptors on antigen presenting cells 22. Our previous works have demonstrated that Bla g 2 contains complex glycans, many of which are mannose terminated, and that they play a critical role in its interaction with the Bla g 2‐mannose receptor (MRC1) in cockroach allergen induced allergic immune responses 23. MRC1 encodes the mannose receptor C‐type lectin, a cell surface protein that belongs to a family of C‐type lectin receptors (CLRs). Several other CLRs, such as dendritic cell‐specific intercellular adhesion molecule‐3‐grabbing non‐integrin receptor (DC‐SIGN) and dendritic cell immunoreceptor (DCIR), have demonstrated recognition for particular glycan moieties on various pathogens and facilitate their endocytosis and presentation as pathogens 22, 24, 25, 26, 27. However, little is known about the function of glycans on cockroach allergens in both antibody‐mediated responses and CLR‐mediated allergen recognition and modulation of the immune response.

In the present study, we provide further evidence that N‐glycans from Bla g 2 are complex hybrid glycans that are terminated with mannose‐, galactose‐, and/or N‐acetyl glucosamine. Moreover, we demonstrate that N‐glycan is critical in IgE antibody binding and the induction of histamine release from basophils. Furthermore, N‐glycan purified from Bla g 2 is able to inhibit cockroach allergic serum‐induced histamine release and IL‐4 production. Importantly, while no MRC1 is expressed on basophils, we provide evidence supporting a critical role of DCIR on the human basophil in mediating cockroach allergen uptake.

## Methods

### Protein and glycoprotein staining

Cockroach whole body extract from *Blattella germanica* (Greer) was separated by SDS–PAGE and stained for total protein using Colloidal Coomassie Blue (Thermo Fisher, Waltham, MA, USA) as directed by the manufacturer. Glycoproteins were stained using the periodic acid‐Schiff stain as described elsewhere 28. Images were then taken of the gel and quantified using ImageJ, Bethesda, MD, USA v1.49u (NIH).

### Protein immobilization, N‐glycan release, and de‐glycosylation of native Bla g 2

Purified Bla g 2 (Indoor Biotechnology, Charlottesville, VA, USA) was immobilized and coupled to a solid support by Glycoprotein Immobilization for Glycan extract (GIG) as previously described 29, 30. In brief, native Bla g 2 was immobilized on Aminolink resin in 500 μL of binding buffer (100 mM sodium citrate and 50 mM sodium carbonate) with the addition of 400 μL of 1 M p‐toluidine in 1 N HCl with 40 μL EDC (N‐[3‐dimethylaminopropyl]‐N′‐ethylcarbodiimide) and 25 μL concentrated HCl. After immobilization, the active aldehyde sites on Aminolink resin was blocked with 50 mM NaCNBH_3_ in 1 M Tris–HCl; 30 min, p‐toluidine was added and the sample was incubated at room temperature for 4 h. The resin was washed with 1% formic acid, 10% ACN (0.1% TFA), 1 M NaCl, and DI water. To release N‐glycan from resin immobilized Bla g 2, the resin was treated with PNGase F (NEB) overnight at 37°C. The supernatant was collected and further purified with Carbograph cartridge 31. A 500 μL solution consisting of 80% ACN in 0.1% TFA was used to elute N‐glycans from the Carbograph cartridge. The eluted sample was dried in a vacuum (Savant SPD SpeedVac) for MALDI‐MS (Shimadzu Resonance Axima, Kyoto, Japan). In contrast, to deglycosylate native Bla g 2, PNGase F was immobilized on Aminolink resin as described above. The resin was incubated with native Bla g 2 in GlycoBuffer 2 (NEB) and incubated at 37°C overnight. The reaction was passed through a Carbograph cartridge and the eluted samples (containing deglycosylated Bla g 2) were dried in a vacuum (Savant SPD SpeedVac) and then re‐suspended in PBS.

## MALDI‐MS

*N*,*N*‐dimethylaniline (4 μL) was added to 200 μL DHB (2,5‐dihydroxybenzoic acid) (prepared by dissolving 200 μg DHB in 200 μL solution consisting of 0.1 mM NaCl in 50% ACN). The N‐linked glycans were dissolved in 40 μL DI in 0.2% TFA, in which 5 μL sample was deposited on μ‐Focus MALDI plate together with 1 μL of DHB‐DMA matrix. The laser power was set to observe typical glycan profiles using either human serum or bovine fetuin with minimum glycan fragmentation in MS1 30. Mass range was focused on m/z 850–2200, respectively, while the average MS spectrum was acquired from 400 MS profiles using Shimadzu Biotech Launchpad (version 2.9.1). Glycan assignment was based on accurate mass, GlycoWorkBench 32, and the Consortium of Functional Glycomics (CFG).

### Cockroach allergic subjects

De‐identified serum samples used in this study were provided by the Johns Hopkins University Dermatology, Allergy and Clinical Immunology (DACI) Reference Laboratory directed by Dr. Robert G. Hamilton. Information for these study subjects are presented in Table 2. All studies presented in these studies were reviewed and approved by the Johns Hopkins University Institution Review Board.

### IgE antibody measurement by solid‐phase binding assay

Solid‐phase binding assays were performed using EIA/RIA 96‐well flat bottom plate (Costar, Columbia, MD, USA) coated with 0.5 μg/mL of CRE, glycosylated, or deglycosylated Bla g 2 in PBS overnight at 4°C. The plate was blocked with 5% w/v bovine serum albumin in TBST (50 mM Tris, 150 mM NaCl, and 0.05% v/v Tween 20) for 1 h and probed with undiluted serum from German cockroach allergic donor subjects for 4 h. The plate was washed and bound IgE antibody was detected using biotinylated monoclonal anti‐human IgE and streptavidin‐horseradish peroxidase. Optical density at 480 nm was measured after the addition of 3,3′,5,5′‐tetramethylbenzidine and H_2_O_2_.

### Cell preparation and culture

Venous blood specimens were anti‐coagulated with EDTA and subjected to double Percoll density centrifugation, as described elsewhere 33. The isolated basophils were quantified by Alcian blue stain and cultured in media consisting of Iscove's modified Dulbecco's medium supplemented with 5% FCS, nonessential amino acids, and 10 µg/mL gentamicin (C‐IMDM).

### Basophil sensitization and challenge

Surface‐bound IgE from donor basophils was removed using cold lactic acid solution (pH, 3.9) 34, washed, and passively sensitized for 30 min with total IgE levels of 500 ng/mL from cockroach allergic serum (as determined by ImmunoCap, Thermofisher Scientific, Halethorpe, MD, USA). It was then challenged with 100 ng/mL of Bla g 2 or deglycosylated Bla g 2 in C‐IMDM in a humidified incubator at 37°C, 5% CO_2_. Cultures were performed in a total volume of 250 mL. Histamine release was measured after 30 min by taking the top 0.05 mL of culture supernatant, diluting in 1 mL acid solution for overnight protein precipitation, and assaying using automated fluorimetry as previously described 34. IL‐4 protein was measured after 4 h incubation by taking the remaining cell‐free supernatant and assaying by ELISA using the Read‐Set‐Go! ELISA set (eBioscience, San Diego, CA, USA).

### Flow cytometry

Expression of C‐type lectin receptors (CLRs) on basophils was detected by means of flow cytometry using a FACS Calibur cytometer (BD Biosciences, San Jose, CA, USA) with α‐CD206 (clone 19.2), α‐DC‐SIGN (clone eB‐h209) and α‐DCIR (clone 9E8), or α‐dectin 1 (clone 15E2) antibodies (eBioscience). The data collected were analyzed with Flowjo software (Treestar, Ashland, OR, USA) 8.

### Binding of cockroach allergen to DCIR

EIA/RIA 96‐well flat bottom plates (Costar) were coated in duplicate with 10 ug/mL of CRE, mannan‐BSA, or BSA for 18 h at 4°C, blocked with 1% BSA in TBST with 100 ug/mL of CaCl_2_ for 1 h at room temperature. Purified recombinant human DCIR‐6xHis (Sino Biological, Beijing, P.R.China) dissolved in blocking buffer was incubated for 1 h at room temperature, washed, and incubated with horseradish peroxidase‐conjugated anti‐6xHis (Santa Cruz Biotechnology, Santa Cruz, CA, USA). The absorbance was recorded at 450 nm in an ELISA reader (BioRad, Hercules, CA, USA).

### Uptake of cockroach allergen by basophils through DCIR

For allergen uptake assays, the purified native Bla g 2 was labeled with Lightning‐Link FITC Antibody Labelling Kit (Odenton, MD, USA). Different doses of FITC labeled purified Bla g 2 were co‐incubated with the cultured basophils (2 × 10^5^) for 1 h at 37°C. Bla g 2 uptake was detected by immunofluorescence staining and flow cytometry, an isotype IgG2a was used as a control.

### Statistical analysis

Statistical significance for normally‐distributed samples was assessed using an independent two‐tailed Student's *t*‐test by using GraphPad Prism version 5.1 software (GraphPad Prism, La Jolla, CA, USA). Data are expressed as the means ± SEM for each group. Differences with *P *< 0.05 were considered statistically significant.

## Results

### Characterization of cockroach glycoproteins

**Figure 1:**
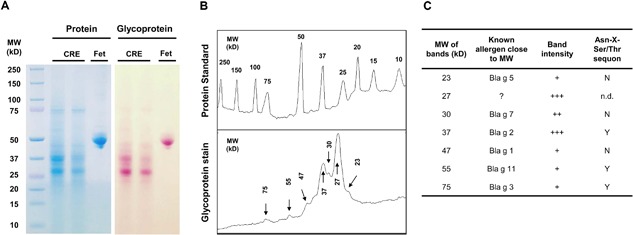
Characterization of cockroach glycoproteins. (A) Glycosylated allergens in two batches of cockroach extract (CRE) were assessed by Coomassie Brilliant Blue and periodic acid‐Schiff staining. Fetuin (Fet) was used as a glycosylated blood protein positive control. (B) Densitometric analysis of the visualized glycoprotein band (peak) was performed using ImageJ v1.49u. (C) Comparison between glycoprotein bands and known cockroach allergens according to the molecular weight (MW). “?” Indicates un‐identified cockroach allergens. Both glycoprotein band intensity and glycosylation Asn‐X‐Ser/Thr sequon are also listed. The intensity of the bands was visually scored as +++ high; ++ medium; and + low. The presence of N‐linked glycosylation Asn‐X‐Ser/Thr sequon was expressed as “Y” (yes), “N” (No), or “n.d” (not determined).

To define the glycosylated proteins in cockroach extract (CRE), CRE was separated on SDS–PAGE. Both proteins and glycoproteins were assessed by Coomassie Brilliant Blue and periodic acid‐Schiff staining, respectively (Fig. 1A). Similar to fetuin (Fet), a control for the glycosylated blood protein, several major bands were identified in CRE. When these visualized glycoprotein bands were quantified by densitometric analysis with ImageJ v1.49 (Fig. 1B), several peaks were noted at approximate MWs of 27, 37, 47, 55, and 75 KD. These prominent bands correspond with known cockroach allergens such as Bla g 2 (37 KD) 35, Bla g 3 (75 KD) 36, and Bla g 11 (55 KD) (unpublished) with one or multiples Asn‐X‐Ser/Thr N‐glycosylated sequon (Fig. 1C). These data provide evidence that the cockroach extract contains multiple glycosylated proteins.

### Profiling of N‐glycan from purified natural Bla g 2 using MALDI‐MS

**Figure 2:**
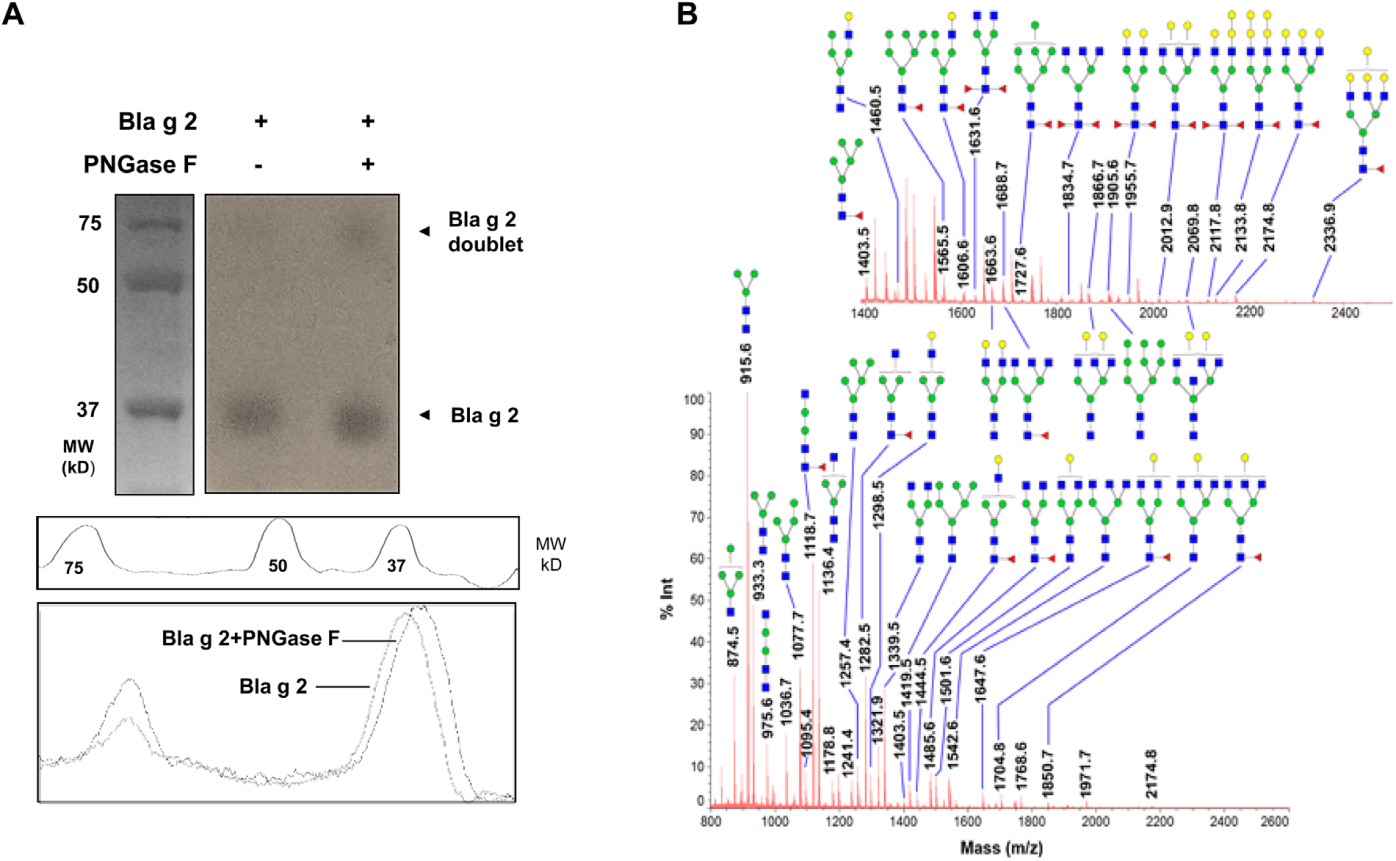
Profiling of N‐glycans from purified natural Bla g 2 using MALDI‐MS: (A) N‐glycan was isolated by PNGase F with a reduced glycosylated allergen in Bla g 2 (lower shift band). (B) Representation of a mass spectrum of Bla g 2 N‐glycans with a mass range that is focused on 850–2200 Da, respectively. Glycan assignment was based on accurate mass‐to‐charge (m/z, X axis) from the consortium of functional glycomics (CFG). Y axis: abundance of a particular glycan as determined by percentage of signal intensity (% Int). (◼) N‐acetylglucosamine, (●) galactose, (●) mannose, (▸) fucose. Glycan masses are reported as mass‐to‐charge ratio (m/z).

To determine the importance of N‐glycans in cockroach allergen, we investigated the N‐glycan structure of purified Bla g 2. N‐glycan was cleaved by PNGase F and a shifted glycosylated allergen in Bla g 2 was observed at an approximate MW of 37 (lower band) (Fig. 2A). N‐glycans were analyzed by MALDI‐MS (Fig. 2B). A total of 51 distinct N‐glycan fragments were identified in a mass range of m/z 850–2200 from the average of 400 mass spectra profiles using Shimadzu Biotech Launchpad, Kyoto, Japan (version 2.9.1) (Table S1). These identified N‐glycans were of two general types: complex and hybrid, with core fucose oligosaccharide modifications. In particular, these complex N‐glycans with the core fucose oligosaccharide modifications are ubiquitously found in plants and insects (i.e. cockroaches) 37. For example, the N‐glycans at m/z 1914.7, 1834.7, 2279.8, and 2361.9 contain di‐fucose moieties linked to N‐acetyl‐glucosamine residues. In addition, many of these N‐glycans terminated with galactose (32/51). Among these, several identified glycans have shown unique terminated carbohydrate moieties and associations with allergic immune responses 23, 26, 38, 39, 40 (Table 1). Together, these data indicate that several major N‐glycans in Bla g 2 may be involved in cockroach allergen‐induced allergic responses.

**Table 1:** Major structures of N‐glycan identified by both MS

Glycan type	Predicted structure	m/z
High mannose	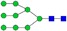	1905.6
Galactose‐terminated	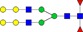	2279.8
	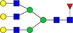	2174.8
N‐acetylglucosamine‐terminated	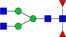	1631.6
	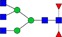	1834.7
Bisecting GIcNAc and complex‐type N‐glycan	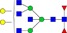	2361.9
Hybrid‐type	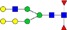	1914.7

### Deglycosylated Bla g 2 had a reduced IgE binding and histamine release in basophils

**Figure 3:**
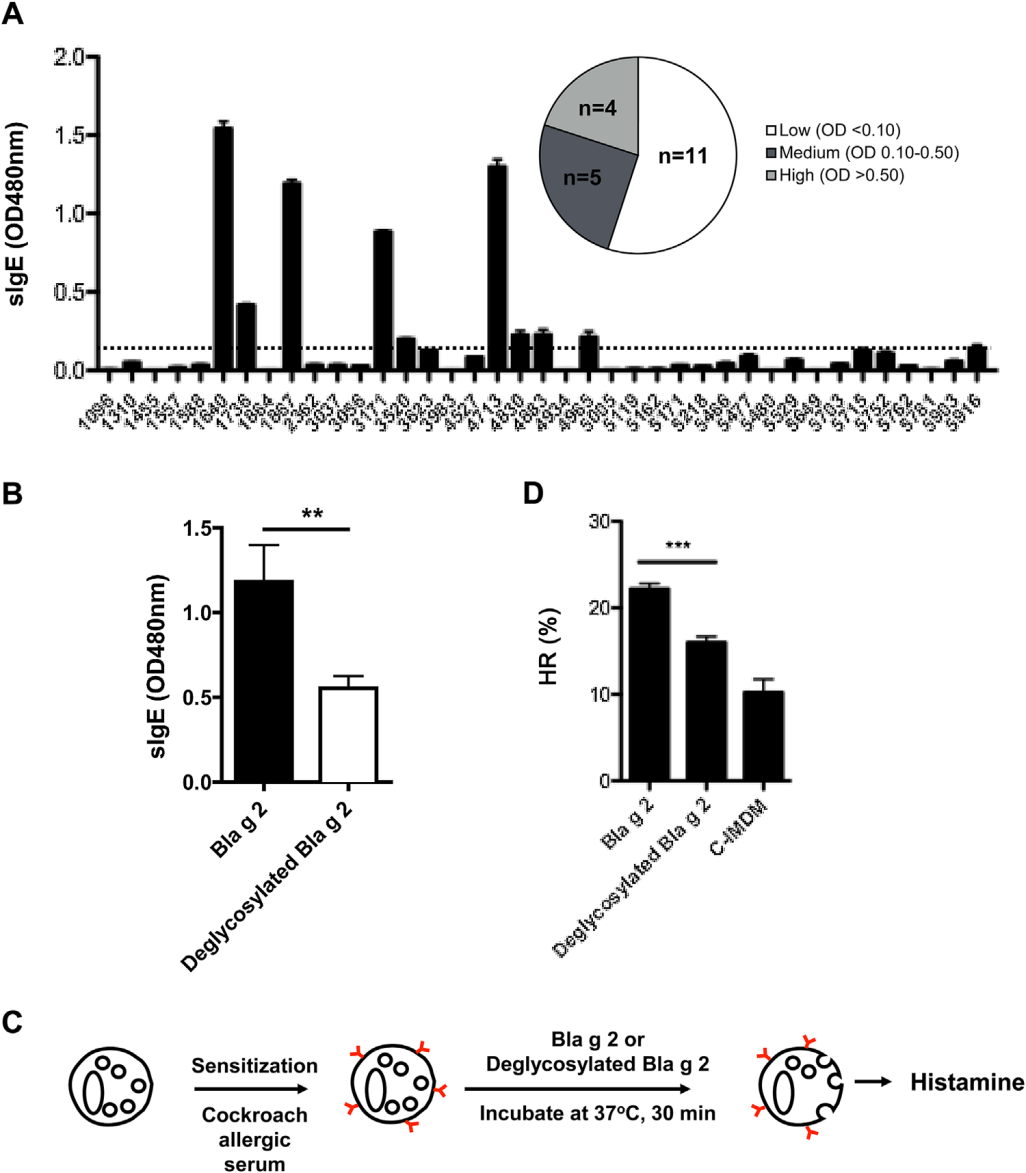
Cockroach allergen specific IgE binding and histamine release for Bla g 2 with or without N‐glycan. (A) Serum from cockroach allergic subjects (*n* = 39) were tested for the levels of Bla g 2 specific IgE (sIgE, OD 480 nm) using ELISA. Dash line indicates mean (2SEM). 1096 was used as a negative control (non‐allergic). (B) Bla g 2 specific IgE antibody was measured using Bla g 2 with (glycan‐Bla g 2) or without glycan (no glycan‐Bla g 2) using sera from cockroach allergic subjects (A) with higher levels of specific IgE. (C) Experimental protocol testing the effect of glycan on histamine release (HR). (D) Histamine release (HR) from basophils that were sensitized with cockroach allergic serum and challenged with Bla g 2 or deglycosylated Bla g 2. C‐IMDM: conditioned Iscove's Modified Dulbecco's Medium. Data represent mean ± SEM. **P < 0.01, ***P < 0.001.

To determine the role of N‐glycans in cockroach allergen‐induced allergic responses, we examined the binding of Bla g 2 or deglycosylated Bla g 2 to German cockroach specific IgE antibody. We quantified the levels of Bla g 2 specific IgE (sIgE) in a total of 39 subjects who are allergic to cockroach allergen (Table 2). Among these, serum from 9 subjects had higher levels of specific IgE antibodies (e.g. OD > 0.10, Fig. 3A). We next selected those subjects with higher levels of sIgE and examined the difference in binding capability of Bla g 2 or deglycosylated Bla g 2 to those specific IgE antibodies (Fig. 3B). Compared to glycosylated Bla g 2, deglycosylated Bla g 2 showed a significant reduction in the levels of sIgE binding. To confirm the importance of glycan in IgE‐mediated responses, we passively sensitized human basophils with serum from subject 1867 and challenged with either glycosylated or deglycosylated Bla g 2 (Fig. 3C). We observed that deglycosylated Bla g 2 induced significantly less histamine release compared to glycosylated Bla g 2 (Fig. 3D). Taken together, these results indicate that N‐glycan is important for the interaction of IgE antibody with cockroach allergen.

**Table 2:** Characteristics of cockroach allergic subjects used for this study (*n* = 39)

ID	Age	CRE slgE	Sex
1096	8	33.90	M
1310	4	45.20	M
1455	44	17.40	M
1557	9	24.20	M
1588	11	11.20	M
1640	12	17.70	M
1736	17	54.90	F
1864	3	25.10	F
1867	12	61.40	F
2362	7	16.50	M
3037	12	34.00	M
3056	6	71.80	F
3171	12	73.90	M
3520	12	13.50	F
3623	6	63.90	M
3983	2	15.70	M
4527	n.r.	2.29	M
4713	5	41.20	M
4830	41	4.28	F
4833	21	27.60	F
4934	14	4.05	M
4965	34	10.60	F
5005	23	0.98	M
5119	15	0.54	F
5218	16	0.77	M
5466	59	0.73	F
5477	13	1.33	M
5480	28	5.94	F
5529	38	6.52	M
5649	26	1.11	F
5703	63	1.41	F
5715	58	0.78	F
5752	45	1.10	F
5762	15	2.84	F
5781	6	1.52	F
5903	3	0.67	M
5916	n.r.	1.34	M

CRE slgE titers are reported as KUA/L as determined by ImmunoCAP (UniCAP; Phadia, Uppsala, Sweden), n.r., not reported; F, female; M, male. All subjects are atopic.

### N‐glycan inhibits spontaneous histamine release

**Figure 4:**
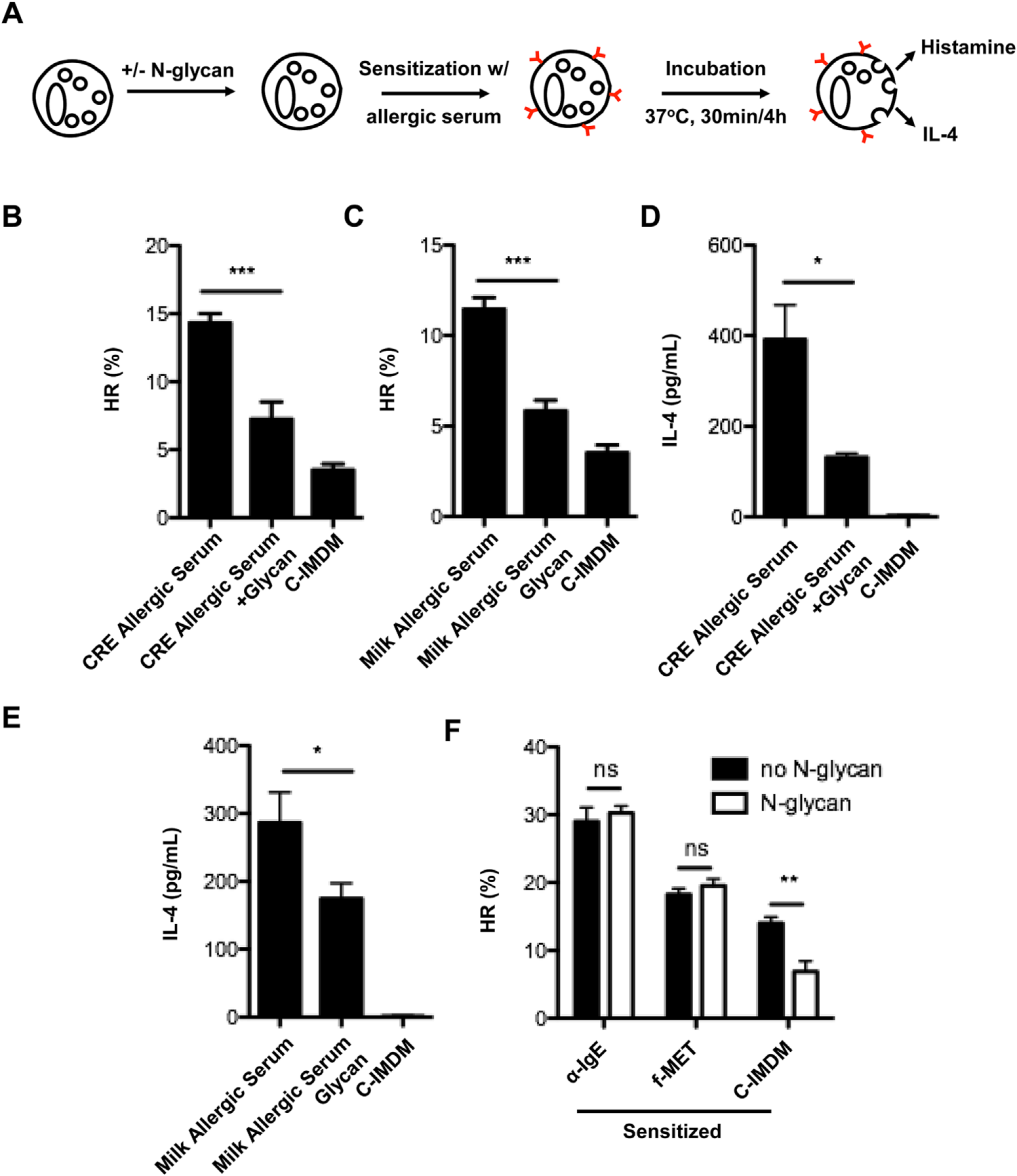
N‐glycan inhibits cockroach allergic serum induced the activation of basophils. (A) Experimental approaches to study the effect of pre‐treated basophils with N‐glycan on histamine release (HR) and IL‐4 secretion. Basophils were stripped of their bound IgE with cold lactic acid, treated with or without N‐glycan, and then passively sensitized with serum from allergic individuals at 37°C for 30 min. (B and C) Histamine release from basophils sensitized with serum from cockroach allergic (CRE, B) and milk allergic (CRE, C) subjects. %HR: percentage of total histamine from equivalent number of basophils. (D and E) levels of IL‐4 from basophils sensitized with serum from cockroach allergic (CRE, D) and milk allergic (CRE, E) subjects. (F) Histamine release from sensitized basophils with α‐IgE or fMet with/without N‐glycan. Data represent mean ± SEM. *P < 0.05, **P < 0.01, ***P < 0.001.

Given that complex carbohydrate has been shown to have immune modulatory properties 41, 42, we tested whether N‐glycan could modify histamine release from basophils using the experimental approach described in Figure 4A. Interestingly, we found that N‐glycan significantly inhibited the spontaneous histamine release from basophils sensitized with serum from cockroach allergic subjects (Fig. 4B). Interestingly, similar results were found when serum from milk allergic subjects was used (Fig. 4C). Furthermore, IL‐4 production, which typically correlated with IgE‐dependent histamine release, but is generated de novo 1–4 h after degranulation, was also measured. Consistent with histamine release, we found that N‐glycan significantly inhibited IL‐4 secretion spontaneously released from basophils passively sensitized by serum from cockroach (Fig. 4D) and milk allergic subjects (Fig. 4E). However, N‐glycan did not affect histamine release in basophils activated by either α‐IgE or N‐Formylmethionine (fMET) or by IgE cross‐linking (Fig. 4F). Taken together, these results support the conclusion that N‐glycan can alter basophil function by suppressing spontaneous histamine release and IL‐4 production.

### Expression of C‐type lectin receptor on human basophil

**Figure 5:**
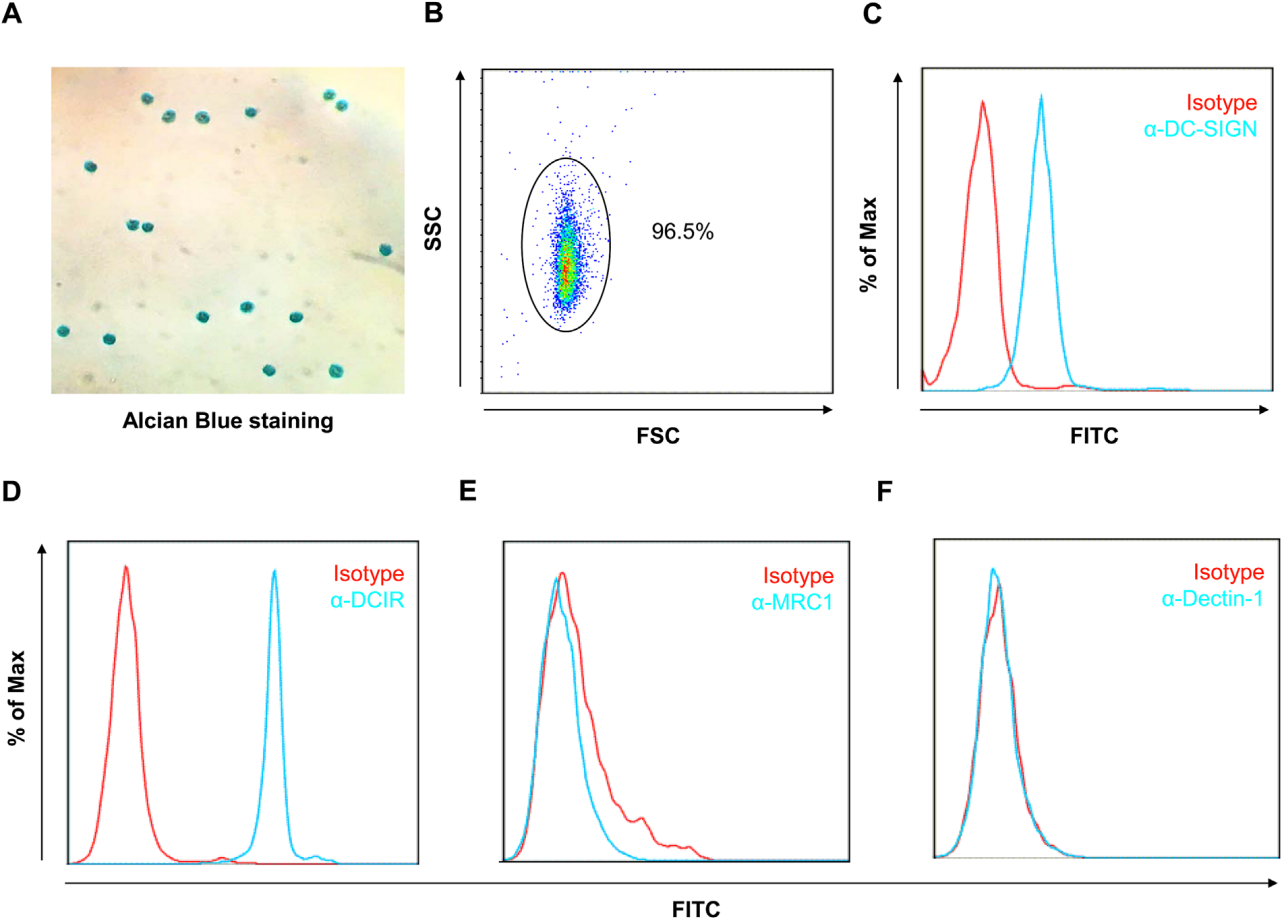
C‐type lectin receptor expression on basophils. (A and B) Basophils were isolated from the peripheral blood and assessed by Alcian Blue staining (A) with >%95 purity (B). (C–F) Expression of CLRs on basophils was determined by flow cytometry, including DC‐SIGN (C), DCIR (D), MRC1 (E), and dectin‐1 (F). IgG was used as an isotype control.

Glycan‐lectin interactions have been shown to be important for allergen‐uptake and the induction of allergen‐induced Th2 responses 22. We detected several major C‐type lectin receptors (CLRs) on human basophils. Basophils were isolated from the blood of human donors and selected with Alcian Blue staining (Fig. 5A) to >%95 purity (Fig. 5B). CLR expression on these isolated basophils was assessed by flow cytometry. DC‐SIGN (Fig. 5C) and DCIR (Fig. 5D) were significantly expressed on human basophils. In contrast, there was no expression for MRC1 (Fig. 5E) and dectin‐1(Fig. 5F). These data indicate that DC‐SIGN and DCIR may be major CLRs contributing to the mediation of allergen‐induced basophil activation.

### DCIR mediates cockroach allergen uptake by human basophils

**Figure 6:**
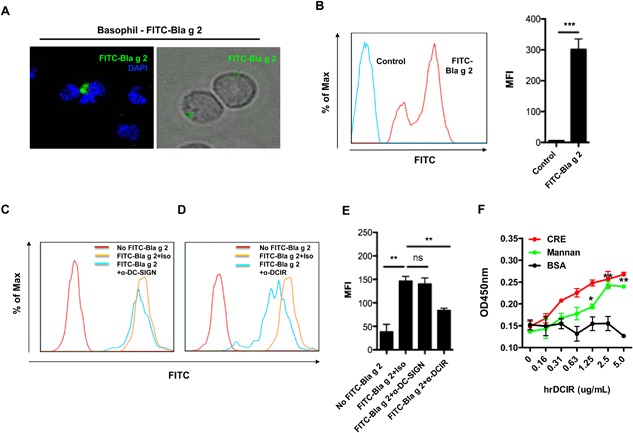
DCIR binds cockroach allergen and mediates FITC‐labeled Bla g 2 uptake by human basophils. (A) Human basophils were incubated with FITC conjugated Bla g 2 (FITC‐Bla g 2, green) for overnight at 37°C, washed, and analyzed by fluorescent microscopy. Nucleus was stained with the DAPI (blue). (B) FITC‐Bla g 2 uptake by basophils was detected by flow cytometry analysis and quantified data represent mean ± SEM. (C) FITC‐Bla g 2 uptake by basophils was detected by flow cytometry when cells were pre‐incubated with isotype control or α‐DC‐SIGN (C) or α‐DCIR (D) for 1 h at 37°C. (E) Quantified data represent mean ± SEM. (F) Binding assay of cockroach allergen and soluble DCIR was performed by coating cockroach extract (CRE), Man‐BSA, and BSA with a serial dilution of 5 μg/mL and then probing with DCIR. *P < 0.05, **P < 0.01, ***P < 0.001.

CLRs expressed on basophils may be involved in mediating allergen‐uptake. Indeed, we found that FITC‐Bla g 2 was taken up by basophils as determined by immunostaining (Fig. 6A). The Bla g 2 uptake by basophils was further confirmed by flow cytometry analysis (Fig. 6B). To determine whether cockroach allergen uptake is dependent on surface CLRs, we pre‐treated basophils with either α‐DC‐SIGN or α‐DCIR antibody, and then co‐cultured with FITC‐Bla g 2 at 37°C for 30 min. While no clear reduction in FITC‐Bla g 2 uptake was observed for basophils pre‐treated with α‐DC‐SIGN (Fig. 6C and E), a significant reduction was observed when basophils were pre‐treated with α‐DCIR (Fig. 6D and E). To further test whether cockroach allergen can bind to DCIR, cockroach extract (CRE) was incubated with different doses of purified recombinant human DCIR (Fig. 6F). Consistent with the Man‐BSA, a positive control, CRE showed a dose‐dependent binding to DCIR. No binding was seen for BSA, a negative control. These results demonstrate that DCIR may be one of the major CLRs that has the capacity to alter allergen‐induced activation of basophils.

## Discussion

Bla g 2 has been identified as one of the most important cockroach allergens as it elicits IgE antibody responses in 40–70% of cockroach allergic individuals 37, 45, 46. Compared to cat and mite allergens, Bla g 2 is able to induce IgE production, even at 10 to 100‐fold lower exposure 43. Furthermore, exposure to Bla g 2 has been linked with wheezing among infants in the first three months of life and with increased T cell proliferation 44. It was assumed that Bla g 2's proteolytic activity contributes to its allergenicity, but structural analysis suggests that Bla g 2 is an inactive aspartic protease 45, 46. Thus, it is important to identify the potential virulence factors in cockroach allergens, particularly in Bla g 2, which could contribute to the development of asthma.

Here, we demonstrate that a number of glycosylated proteins are present in CRE. Interestingly, we found that one of the most prominent glycosylated proteins corresponded to Bla g 2 35. Using glycomic studies, we revealed that Bla g 2 contain N‐glycans that are complex hybrid‐types that are terminated with mannose‐, galactose‐, and/or GlcNAc. While we have previously reported predominately high mannose N‐glycans in Bla g2 23, this work provides further validation and more extensive analyses by providing a comprehensive list of glycans with more complex structures in Bla g 2. As expected, many of these identified N‐glycans are common variants of the core fucose oligosaccharide modifications that are ubiquitously found in plants and insects 37. These glycan structures are distinct from human endogenous glycans. It is likely that some of these glycans may contribute to the allergenicity of cockroach allergen. Indeed, the immunogenicity of some of these identified glycans have been reported in human and mouse models 47, 48. More specifically, glycans terminated with mannose are able to modulate fibrocyte function through interacting with MRC1 23, or regulate myeloid DCs through DC‐SIGN 38, or trigger cysteinyl leukotriene generation via Dectin‐2 49. Glycans terminated with galactose, for example, galactose alpha‐1,3‐galactose (alpha‐gal), have been associated with delayed type anaphylaxis to red meat in patients with α‐Gal specific IgE 39. α‐Gal is a sugar chain commonly found as part of glycoproteins and glycolipids in mammals 50. In addition, glycans terminated with GlcNAc are critical in egg‐white ovalbumin‐induced IgE production and Th2 cytokine secretion 51. Taken together, these findings provide evidence supporting a role of glycans in allergic immune responses to cockroaches.

We next examined the role of N‐glycans in Bla g 2 in bindng IgE antibody using serum from cockroach allergic subjects with high levels of Bla g 2‐specific IgE. Interestingly, we observed a significant reduction in IgE binding to Bla g 2 with the removal of its N‐glycan compared to binding to native glycosylated Bla g 2. These data indicate that N‐glycan may be a critical component involved in IgE antibody binding to Bla g 2. This raises the possibility that N‐glycan present in Bla g 2 is able to induce anti‐glycan IgE responses. Indeed, we found that Bla g 2‐derived N‐glycan alone can directly bind to IgE in the serum from cockroach allergic subjects (Data not shown). This observation is also consistent with previous reports that ∼15–30% of allergic patients produce specific anti‐glycan IgE 52, 53, 54, 55. The core‐3‐linked fucose is one of the most common epitopes in allergens that is recognized by human IgE antibodies 40.

We also investigated whether reduced binding to IgE antibody occurs when N‐glycans are removed from Bla g 2. Moreover, does reduced binding alter the ability of IgE antibody to trigger mediator release from sensitized basophils? We observed that deglycosylated Bla g 2 induced less histamine release when compared to glycosylated Bla g 2. These findings are further supported by recent studies showing that anti‐glycan IgE from allergic patients can induce mediator release from mast cells. Anti‐CCD (cross‐reactive carbohydrate determinant) IgE is also sufficient to trigger mediator release from basophils 17, 56, 57, 58. Collectively, these results suggested that N‐glycan is important for allergen binding to IgE antibody and potentially alter down‐stream mediator release from human basophils.

Given the significance of glycans in allergen binding to IgE antibody and activation of basophils, it was of interest to see whether glycans on their own could contribute to the induction of allergic responses, particularly histamine release from basophils. Basophils from food allergic children have been shown to release histamine “spontaneously” during incubation of suspensions in vitro without the addition of antigen 59. We therefore pre‐treated basophils with N‐glycan directly to see whether glycan could induce spontaneous histamine release as well as that induced by allergen. Unexpectedly, basophils treated with glycan spontaneously released much lower levels of histamine when compared with those that were un‐treated (Data not shown), suggesting that glycan may have a suppressive role in this response. In contrast, allergen‐induced histamine release was not affected by pretreatment with glycan. It's recently been reported that spontaneous histamine is transferable to basophils of non‐allergic subjects following passive sensitization with serum from allergic subjects—a reaction dependent on IgE 60. We further confirmed this finding by using basophils passively sensitized with serum from a cockroach allergic individual. Whereas spontaneous histamine release was transferred following this sensitization, basophils pre‐treated with glycan released significantly less histamine in medium alone. This finding was further replicated using the same experimental approach but with serum from milk allergic subjects. Like histamine, a similar pattern was observed for IL‐4 in supernatants of these treated basophils. These results indicate that glycan alone can modify basophil function by suppressing spontaneous histamine release and IL‐4 production. However, we did not find the inhibition of histamine release by glycan when basophils were activated by α‐IgE or fMET or IgE‐dependent cross‐linking. We assumed that the dose of glycan used may not have been sufficient to inhibit basophil activation triggered by via IgE cross‐linking. Also, it is likely that the glycan inhibition may be through non‐IgE‐mediated signaling pathways such as the glycan‐CLR axis.

CLRs are abundantly expressed on the surface of antigen‐presenting cells 61. Lectin‐carbohydrate interactions are critical in pathogen recognition and stimulation of innate immune responses and it can modulate glycan immunogenicity 22, 62. For instance, glycans in house dust mite extract induce Th2 differentiation through interaction with Dectin‐2 on DCs and lead to the generation of cysteinyl leukotrienes 49. Our previous studies also demonstrated that MRC1 on fibrocytes mediate cockroach allergen binding, internalization, and down‐stream immune reactions 23. To determine the role of CLRs in basophils, we analyzed several major CLRs that have been reported for their interactions with glycans terminated with mannose (MRC1 23), galactose (DC‐SIGN 38), and/or GlcNAc (Dectin‐2 49). Another important CLR, DCIR, was also included in our analysis. DCIR, containing immunoreceptor tyrosine‐based inhibitory motifs in their cytoplasmic tails, has been shown to regulate the suppression of TLR‐induced IL‐12 and TNF production by DCs 63, 64, 65. We found that human basophils predominately express DCIR and DC‐SIGN, but not MRC1 and Dectin‐2. Furthermore, DCIR, but not DC‐SIGN, mediates cockroach allergen‐uptake by basophils, suggesting that DCIR may be one of the major CLRs contributing to the allergen‐induced activation of basophils.

Collectively, we report that Bla g 2 contains glycans with complex structures, and that N‐glycan is critical in IgE binding and histamine release from basophils. Furthermore, we have demonstrated, for the first time, that N‐glycan purified from Bla g 2 has a suppressive role in spontaneous histamine release and IL4 production from basophils sensitized with cockroach allergic serum. Importantly, DCIR may serve as a target for future investigation into its role in mediating allergen recognition, particularly for selected glycans that may modulate basophil function. As a result, these studies suggest a new conceptual framework for the role of glycan in allergen‐induced allergic responses by linking the glycan/allergen‐DCIR axis to basophil activation and development of allergic disease and asthma.

## Author Contributions

PG and DCD designed experiments. DCD performed the experiments and analyzed the data. SY, XY, RGH, and JTS helped with the experiments and data analysis. PG and DCD wrote the manuscript.

## Conflict of Interest

None declared.

## Supporting information

Additional supporting information may be found in the online version of this article at the publisher's web‐site.

**Table S1:** Predicted structures of N‐glycan from Bla g 2.

**Table S2:** List of N‐glycans from Bla g 2 identified by Glycoprotein Immobilization for Glycan Extraction followed by MALDI‐MS
