# Supplementary material for: N‐glycan in cockroach allergen regulates human basophil function
Source: Immun Inflamm Dis. 2017 Feb 20;5(4):386–99. doi: 10.1002/iid3.145 (PMC5691304; doi:10.1002/iid3.145)
Supplement: Supplementary file 1 — Table S1: Predicted structures of N‐glycan from Bla g 2. [file IID3-5-386-s001.pptx]

## Slide 1
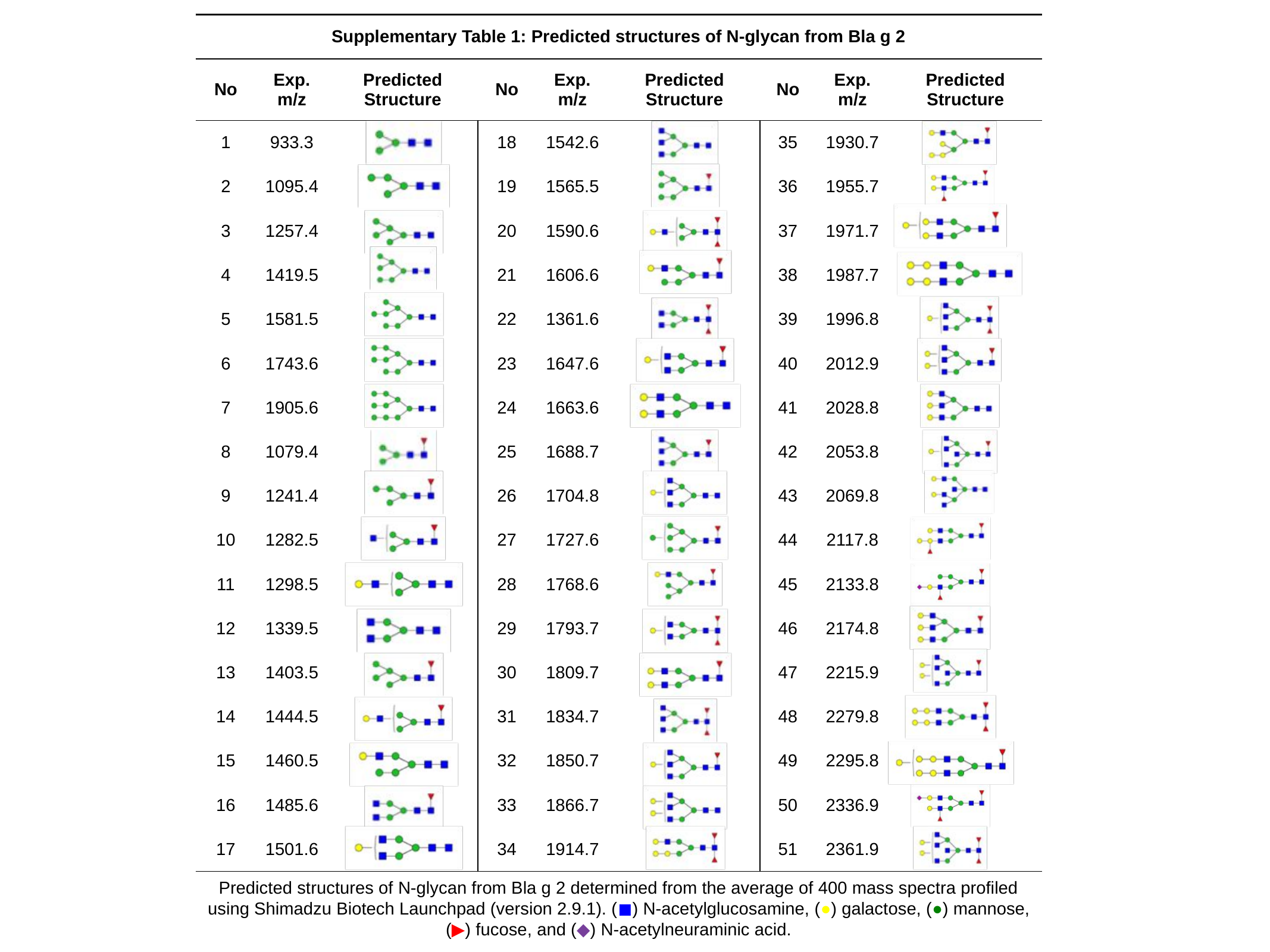

| Supplementary Table 1: Predicted structures of N-glycan from Bla g 2 | | | | | | | | |
| --- | --- | --- | --- | --- | --- | --- | --- | --- |
| No | Exp. m/z | Predicted Structure | No | Exp. m/z | Predicted Structure | No | Exp. m/z | Predicted Structure |
| 1 | 933.3 | | 18 | 1542.6 | | 35 | 1930.7 | |
| 2 | 1095.4 | | 19 | 1565.5 | | 36 | 1955.7 | |
| 3 | 1257.4 | | 20 | 1590.6 | | 37 | 1971.7 | |
| 4 | 1419.5 | | 21 | 1606.6 | | 38 | 1987.7 | |
| 5 | 1581.5 | | 22 | 1361.6 | | 39 | 1996.8 | |
| 6 | 1743.6 | | 23 | 1647.6 | | 40 | 2012.9 | |
| 7 | 1905.6 | | 24 | 1663.6 | | 41 | 2028.8 | |
| 8 | 1079.4 | | 25 | 1688.7 | | 42 | 2053.8 | |
| 9 | 1241.4 | | 26 | 1704.8 | | 43 | 2069.8 | |
| 10 | 1282.5 | | 27 | 1727.6 | | 44 | 2117.8 | |
| 11 | 1298.5 | | 28 | 1768.6 | | 45 | 2133.8 | |
| 12 | 1339.5 | | 29 | 1793.7 | | 46 | 2174.8 | |
| 13 | 1403.5 | | 30 | 1809.7 | | 47 | 2215.9 | |
| 14 | 1444.5 | | 31 | 1834.7 | | 48 | 2279.8 | |
| 15 | 1460.5 | | 32 | 1850.7 | | 49 | 2295.8 | |
| 16 | 1485.6 | | 33 | 1866.7 | | 50 | 2336.9 | |
| 17 | 1501.6 | | 34 | 1914.7 | | 51 | 2361.9 | |
Predicted structures of N-glycan from Bla g 2 determined from the average of 400 mass spectra profiled using Shimadzu Biotech Launchpad (version 2.9.1). (◼︎) N-acetylglucosamine, (●) galactose, (●) mannose, (▶︎) fucose, and (◆) N-acetylneuraminic acid.
