# Supplementary material for: N‐glycan in cockroach allergen regulates human basophil function
Source: Immun Inflamm Dis. 2017 Feb 20;5(4):386–99. doi: 10.1002/iid3.145 (PMC5691304; doi:10.1002/iid3.145)
Supplement: Supplementary file 2 — Table S2: List of N‐glycans from Bla g 2 identified by Glycoprotein Immobilization for Glycan Extraction followed by MALDI‐MS [file IID3-5-386-s002.pptx]

## Slide 1
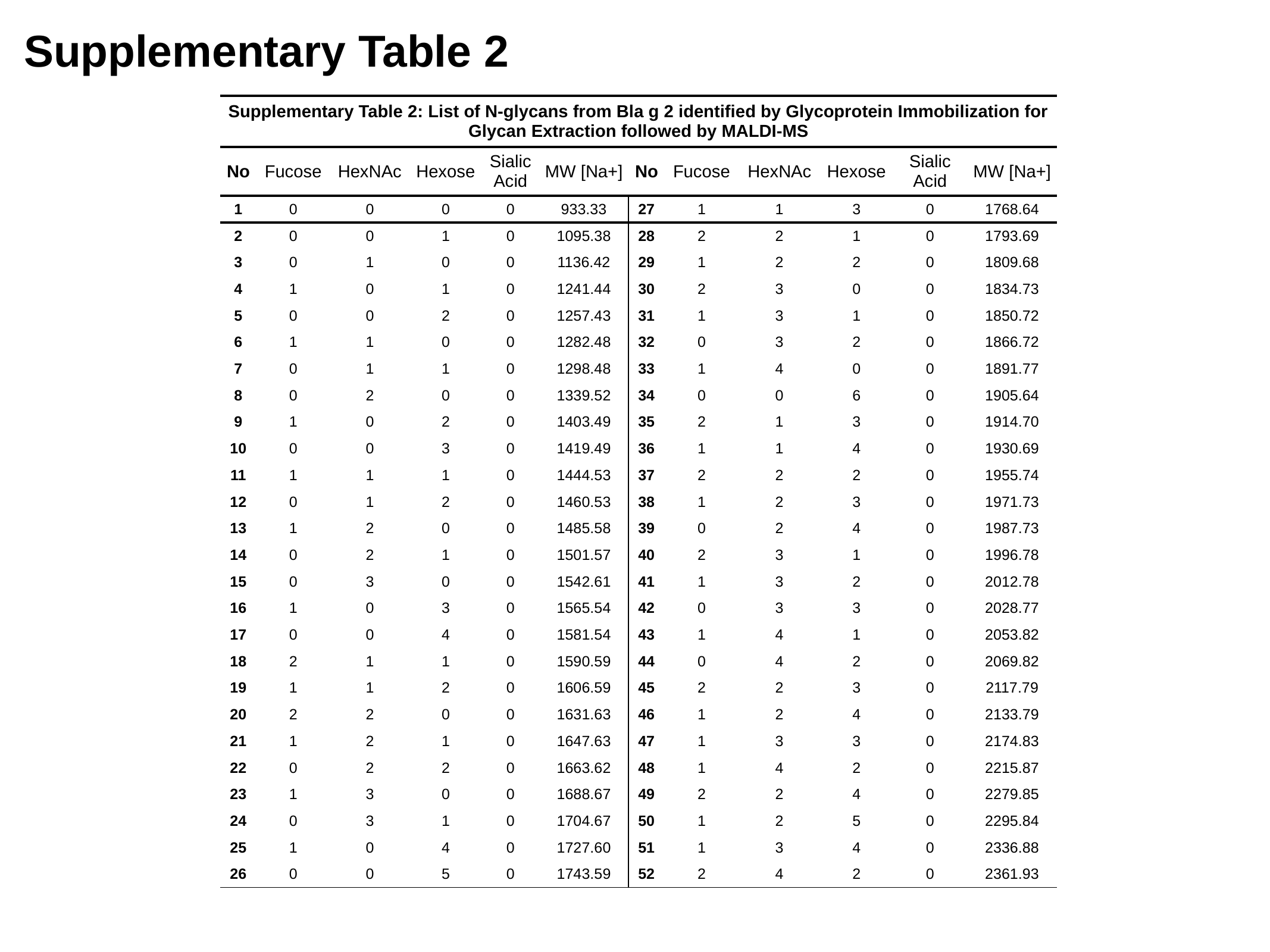

Supplementary Table 2
| Supplementary Table 2: List of N-glycans from Bla g 2 identified by Glycoprotein Immobilization for Glycan Extraction followed by MALDI-MS | | | | | | | | | | | |
| --- | --- | --- | --- | --- | --- | --- | --- | --- | --- | --- | --- |
| No | Fucose | HexNAc | Hexose | Sialic Acid | MW [Na+] | No | Fucose | HexNAc | Hexose | Sialic Acid | MW [Na+] |
| 1 | 0 | 0 | 0 | 0 | 933.33 | 27 | 1 | 1 | 3 | 0 | 1768.64 |
| 2 | 0 | 0 | 1 | 0 | 1095.38 | 28 | 2 | 2 | 1 | 0 | 1793.69 |
| 3 | 0 | 1 | 0 | 0 | 1136.42 | 29 | 1 | 2 | 2 | 0 | 1809.68 |
| 4 | 1 | 0 | 1 | 0 | 1241.44 | 30 | 2 | 3 | 0 | 0 | 1834.73 |
| 5 | 0 | 0 | 2 | 0 | 1257.43 | 31 | 1 | 3 | 1 | 0 | 1850.72 |
| 6 | 1 | 1 | 0 | 0 | 1282.48 | 32 | 0 | 3 | 2 | 0 | 1866.72 |
| 7 | 0 | 1 | 1 | 0 | 1298.48 | 33 | 1 | 4 | 0 | 0 | 1891.77 |
| 8 | 0 | 2 | 0 | 0 | 1339.52 | 34 | 0 | 0 | 6 | 0 | 1905.64 |
| 9 | 1 | 0 | 2 | 0 | 1403.49 | 35 | 2 | 1 | 3 | 0 | 1914.70 |
| 10 | 0 | 0 | 3 | 0 | 1419.49 | 36 | 1 | 1 | 4 | 0 | 1930.69 |
| 11 | 1 | 1 | 1 | 0 | 1444.53 | 37 | 2 | 2 | 2 | 0 | 1955.74 |
| 12 | 0 | 1 | 2 | 0 | 1460.53 | 38 | 1 | 2 | 3 | 0 | 1971.73 |
| 13 | 1 | 2 | 0 | 0 | 1485.58 | 39 | 0 | 2 | 4 | 0 | 1987.73 |
| 14 | 0 | 2 | 1 | 0 | 1501.57 | 40 | 2 | 3 | 1 | 0 | 1996.78 |
| 15 | 0 | 3 | 0 | 0 | 1542.61 | 41 | 1 | 3 | 2 | 0 | 2012.78 |
| 16 | 1 | 0 | 3 | 0 | 1565.54 | 42 | 0 | 3 | 3 | 0 | 2028.77 |
| 17 | 0 | 0 | 4 | 0 | 1581.54 | 43 | 1 | 4 | 1 | 0 | 2053.82 |
| 18 | 2 | 1 | 1 | 0 | 1590.59 | 44 | 0 | 4 | 2 | 0 | 2069.82 |
| 19 | 1 | 1 | 2 | 0 | 1606.59 | 45 | 2 | 2 | 3 | 0 | 2117.79 |
| 20 | 2 | 2 | 0 | 0 | 1631.63 | 46 | 1 | 2 | 4 | 0 | 2133.79 |
| 21 | 1 | 2 | 1 | 0 | 1647.63 | 47 | 1 | 3 | 3 | 0 | 2174.83 |
| 22 | 0 | 2 | 2 | 0 | 1663.62 | 48 | 1 | 4 | 2 | 0 | 2215.87 |
| 23 | 1 | 3 | 0 | 0 | 1688.67 | 49 | 2 | 2 | 4 | 0 | 2279.85 |
| 24 | 0 | 3 | 1 | 0 | 1704.67 | 50 | 1 | 2 | 5 | 0 | 2295.84 |
| 25 | 1 | 0 | 4 | 0 | 1727.60 | 51 | 1 | 3 | 4 | 0 | 2336.88 |
| 26 | 0 | 0 | 5 | 0 | 1743.59 | 52 | 2 | 4 | 2 | 0 | 2361.93 |
